# Tuberculosis screening at the Sainte-Anne Hospital in Paris – results of first and second IGRA

**DOI:** 10.1186/1745-6673-9-24

**Published:** 2014-07-08

**Authors:** Albert Nienhaus, Paul-Kenneth Gariepy, Catherine Trouve, Christiane Lhaumet, Jean Toureau, Claudia Peters

**Affiliations:** 1 Center of Excellence for Epidemiology and Health Services Research for Healthcare Professionals (CVcare), University Medical Center Hamburg-Eppendorf (UKE), Hamburg, Germany; 2 Principles of Prevention and Rehabilitation Department (GPR), Institute for Statutory Accident Insurance and Prevention in the Health and Welfare Services (BGW), Hamburg, Germany; 3 Department of Occupational Safety and Health, Sainte-Anne Hospital, Paris, France; 4 Institute for Health Service Research in Dermatology and Nursing, University Medical Center Hamburg-Eppendorf, Martinistrasse 52, 20246 Hamburg, Germany

**Keywords:** Tuberculosis, Healthcare workers, Interferon-gamma release assay, Reversion

## Abstract

**Introduction:**

Healthcare workers (HCWs) are exposed to Mycobacterium tuberculosis (MTB) and therefore are screened for tuberculosis (TB). Results of TB screenings with the Interferon-γ Release Assay (IGRA) in a French psychiatric hospital without a TB ward are described.

**Methods:**

At the Sainte-Anne Hospital, a referral centre for psychiatric patients throughout the municipal region of Paris, IGRA screening is performed during pre-employment and general health examination or after potential contact to MTB. The QuantiFERON Gold in tube (QFT) is used and data on TB history are assessed in a standardized manner.

**Results:**

Between August 2008 und August 2013 in total 1.192 HCWs were tested and the QFT was positive in 265 (22.2%). Probability of a positive QFT increased with age. A second QFT was performed in 144 HCWs with a positive QFT and 53 (36.8%) HCWs had a reversion. With a positive QFT close to the cut-off (e.g. 0.35-0.7 IU/ml) the odds ratio for a reversion was 4.6 compared to an INF-γ concentration of ≥3.0 IU/ml. Probability of reversion was not influenced by preventive chemotherapy, which was completed by 28 (19.4%) HCWs with a positive QFT. No active TB was detected.

**Conclusion:**

Prevalence of positive IGRA is high in French HCWs as is the number of reversions in IGRA. Reversion rate is particularly high around the cut-off of the IGRA. A borderline zone will therefore reduce the influence of test variability.

## Introduction

The risk of contracting tuberculosis (TB) is increased in healthcare workers’ (HCWs) [1-3]. TB screening for HCWs is therefore considered a cornerstone for preventing TB in hospitals [4]. Until now, TB screening was performed using a tuberculin skin test (TST), which has several weaknesses the most important being cross reactivity with BCG vaccination and booster phenomena due to intradermal application. The interferon-gamma release assays (IGRA) are a promising tool to overcome these problems [5-7]. Because IGRA use antigens specific to *Mycobacterium tuberculosis* they do not show cross reactivity with BCG vaccination and most non-tuberculosis mycobacteria. As IGRA are in vitro tests the problem of boosting in serial testing is circumvented. IGRA correlate better than TST with exposure to infectious patients [8]. Furthermore, in low incidence countries IGRA have a higher predictive value for disease progression [9-11]. Therefore IGRA are likely to improve both the effectiveness and the efficiency of HCWs screening [12]. However, interpretation of IGRA in the serial testing of HCWs remains to be clarified and a consensus needs to be found as the reversion rate in positive IGRA seems to be high [7,13-17]. The reversion rate depends on the quantitative result of the positive first IGRA. Around the cut-off the reversion rate is particularly high and the clinical importance of this observation is not jet well understood. Do they reflect transient infection, good control of the infection with no further stimulation of the immune system or just variability by chance [5]? So far we do not have the answer to this question. However it is questionable whether HCWs with a positive but low result in the IGRA should receive preventive treatment or whether they should be retested before a decision is made [15,17].

In France the incidence of TB is low (8.9 cases per 100,000 in the year 2007), however the variability of the incidence rate is high. In the region of Paris the incidence rate is as high as 18.4/100,000. Therefore TB prevention is a public health priority in France [18]. Special focus is given to nosocomial infections and the screening of HCWs [19,20]. Since 2007 the French Health Authority has recommended the use of IGRA for these screenings. Published data concerning the results of TB-screenings with IGRA are available from four studies, reporting prevalence rates of positive IGRA from 12% to 32% while a TST ≥10 mm was observed in 43% to 70% of HCWs [21-24]. However data on reversions in IGRA are still sparse as only one French study covered this subject [24].

At the Sainte-Anne Hospital in Paris TB screening of HCWs with IGRA was started in 2008 and those HCWs with a positive IGRA were offered retesting. Therefore the prevalence of positive IGRA and the reversion rate of positive IGRA can be described.

## Method

The population of this prospective study includes all workers at the Sainte-Anne Hospital in Paris who participated in TB screening between August 2008 and August 2013 due to pre-employment screening, general occupational health (OH) examination or contact with infectious TB patients or materials, and on whom an IGRA was performed. In contact tracings the screening was performed 8 weeks after last contact. The Sainte-Anne Hospital specializes in psychiatry and it is a referral centre for psychiatric patients throughout the municipal region of Paris. The hospital has no TB ward.

Results of the TB screening were assessed in a standardized database. BCG vaccination for all new-borns was mandatory in France until 2008 [19]. Therefore it was assumed that all HCWs were vaccinated, no data on BCG vaccination was considered in this analysis. The TST results in history were assessed from the individual files of the HCWs or by interview. No simultaneous testing with IGRA and TST was performed. All HCWs with a first positive IGRA were retested within 3 months. Only if the second IGRA was positive, too, the respective HCWs was referred to a specialist for consultation concerning preventive chemotherapy of latent TB infection (LTBI).

For the IGRA, the QuantiFERON® -TB Gold In-Tube Assay (QIAGEN, Cellestis) (QFT) was administered in accordance with the manufacturer’s instructions. As the study used anonymous data generated in the scope of routine OH examinations no approval of an ethics committee was needed.

### Statistical analysis

Chi-square tests were used for categorical data. Adjusted odds ratios (OR) and 95% confidence intervals (CI) were calculated for putative predictive variables using conditional logistic regression. Model building was performed backwards using the change criteria for variable selection.

## Results

**Figure 1:**
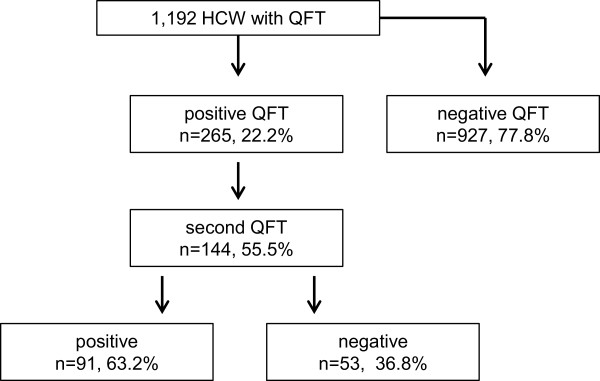
Flow chart of the study population.

**Figure 2:**
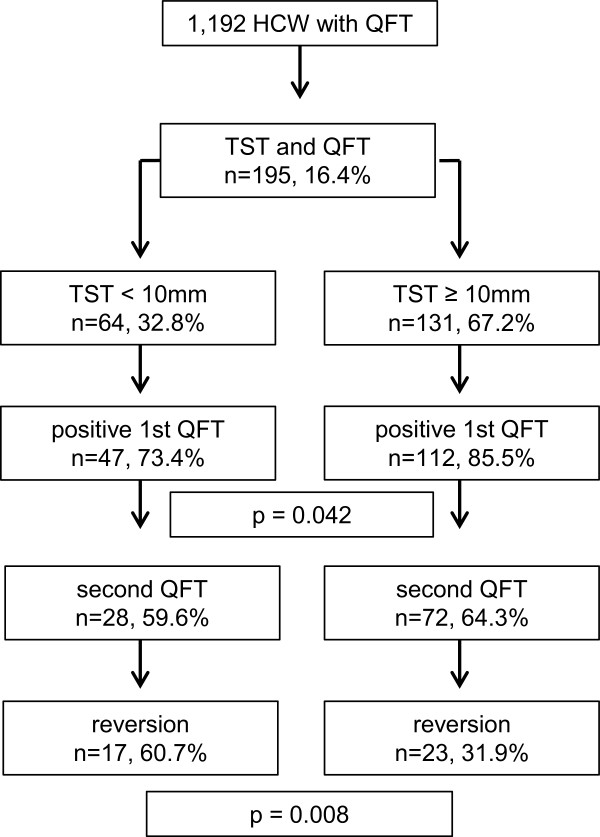
Flow chart of HCWs with a TST in history.

The study population comprises 1.192 HCWs. The study population is described in Table 1. In total 265 (22.2%) of the HCWs were positive in IGRA (Figure 1). No difference was observed between male and female HCWs concerning IGRA results (Table 2). Prevalence of positive IGRA increased with age, if the very young age group (<20 years) is disregarded. No difference was observed regarding the reason for the test, general screening or pre-employment screening. A second IGRA was performed in 144 HCWs with a positive IGRA (55.5%) and a negative second IGRA was observed in 53 HCWs (36.8%). The results of a TST in history were documented for 195 HCWs (16.4%). The TST was below 10 mm in 32.8% and at least 10 mm in 67.2% (Figure 2). The IGRA was more often positive in HCWs with a positive TST (85.5 versus 73.4%, p = 0.04) than in those with a negative TST in history.

**Table 1:** Study population (n = 1.192)

	**N**	**%**
Female	810	68.0
Male	382	32.0
Age in years		
<20	15	1.3
20- < 25	248	20.8
25- < 30	209	17.5
30- < 40	285	23.9
40- < 50	204	17.1
50- < 60	181	15.2
60+	50	4.2
Reason for examination		
General OH examination	393	33.0
Preemployment screening	769	64.5
Contact tracing	30	2.5
First QFT		
Negative	927	77.8
Positive	265	22.2
TST result known	195	16.4
Second QFT performed	144	12.1
Reversion in IGRA	53	36.8*

*out of 144 with a second QFT.

**Table 2:** Risk factors for a positive QFT

	**QFT positive**			
	**N**	**%**	**OR**	**95% CI**
Female	152	19.3	1	--
Male	83	22.3	1.2	0.9 – 1.6
Age in years				
<20	4	26.7	2.2	0.7 – 7.4
20- < 25	34	14.0	1	--
25- < 30	33	16.1	1.2	0.7 – 7.4
30- < 40	55	19.7	1.5	0.7 – 2.0
40- < 50	46	22.8	1.8	1.1 – 3.0
50- < 60	48	28.2	2.4	1.5 – 4.0
60+	15	31.3	2.8	1.4 – 5.7
Reason for examination				
General OH exam	83	21.1	1	--
Pre-employment	152	19.8	1.2	0.9 – 1.7
Contact tracing*	30	100.0	--	

*30 HCWs, who were examined because of a contact tracing, where excluded from the model as all 30 HCWs had a positive QFT.

Reversions were more likely in those with a negative TST in history (60.7 versus 31.9%, p = 0.008). Furthermore reversions were less likely when the IGRA was taken because of a pre-employment screening and more likely when the second IGRA was performed within three months after the first IGRA (OR 2.3; 95% CI 1.2-6.8) (Table 3). If the concentration of the INF-γ was between 0.35 and <0.7 IU/ml the probability of a reversion was increased (OR 4.6, 95% CI 1.6-13.4). If the IFN-γ concentration was at least 3.0 IU/ml in the first QFT, probability of a reversion was still 20%. No active TB was diagnosed in the scope of this study.

**Table 3:** Risk factors for reversion

	**QFT reversion**			
**Reason for examination**	**N**	**%**	**OR**	**95% CI**
General OH exam	26	48.1	1	--
Pre-employment	20	26.7	0.3	0.1 – 0.9
Contact tracing	7	46.7	1.3	0.4 – 4.6
Time between the QFT				
<3 months	28	45.9	2.3	1.2 – 6.8
3+ months	25	30.1	1	--
Concentration of first QFT				
0.35- < 0.7 IU/ml	20	55.6	4.6	1.6 – 13.4
0.7- < 1.0 IU/ml	9	30.6	2.2	0.7 – 7.5
1.0- < 3.0 IU/ml	15	39.5	2.4	0.8 – 7.1
3.0+ IU/ml	9	20.0	1	--
TST in history				
10 + mm	23	31.9	1	
<10mm	17	60.7	4.4	1.6 – 12.2
Unknown, not performed	13	29.5	1.3	0.5 – 3.2

## Discussion

To our knowledge this study is the first to describe a positive association between age and positive IGRA results in French HCWs. Furthermore we observed a high reversion rate of IGRA in HCWs, which was highest when the INF-γ concentration of the first IGRA was between > =0.35 and < 0.7 IU and when the second IGRA was repeated within 3 months after the first IGRA. No active TB was diagnosed within the scope of the study and the prevalence of positive IGRA was lower than the prevalence of positive TST in history.

As most French HCWs are BCG vaccinated the discrepancy between TST and IGRA results are well explained. In so far our study corroborates the observations of other studies [23-25]. A high reversion rate of positive IGRA was reported by other studies before [26-33]. As no active TB cases were observed in our study, information for the clinical interpretation of a reversion in IGRA cannot be derived from our data. As the TB risk in our collective seems to be low, it might be safe to conclude that HCWs with a reversion in IGRA will not profit from preventive chemotherapy. In line with this it was proposed that QFTs with an INF-γ concentration between 0.35 and 1.11 IU/ml should be repeated before any other action (X-ray, referral to expert or chemoprevention) is taken in low risk HCWs [15]. Surprisingly, the reversion rate was higher when the IGRA was repeated within three months of the first IGRA. The delay in repeating a first positive IGRA was based on organizational hinders, which should not have influenced the IGRA results.

As only HCWs with positive IGRA results were retested, no information on conversion rates in French HCWs can be derived from our study. In a German low risk group no conversion was observed when a borderline zone for the QFT of 0.2 to 0.7 IU/ml was applied [34,35]. However, recently several reports were published about higher conversion rates in IGRA than in TST [36-39]. This might indicate that some of the conversions of the IGRA are explained by chance or measurement variability. As IGRA conversion rates are higher when the INF-γ of the first IGRA is close to the cut-off, it seems reasonable to introduce a borderline zone for IGRA interpretation in serial testing or to define a minimum increase in INF-γ concentration that need to be exceeded for a conversion. In addition it seems reasonable to repeat all first time IGRAs which are positive and to perform X-ray for the exclusion of active TB in the absence of clinical symptoms or preventive chemotherapy only in those HCWs with a confirmed positive IGRA.

## Conclusion

Prevalence of positive IGRA is high in French HCWs as is the number of reversions in IGRA. Reversion rate is particularly high around the cut-off of the IGRA. Before chemoprevention is administered the IGRA should be repeated as HCWs with a reversion in IGRA will most likely not profit from preventive treatment. In HCWs with a reversion in IGRA and no clinical symptoms it seems save not to perform an X-ray. More research is needed in order to better understand the variability of IGRA results. Ultimately a more stable IGRA seems to be desirable.

## Competing interests

All authors declare that they have no competing interests.

## Authors’ contributions

AN performed data analysis and wrote the first draft of the manuscript. PKG designed the study, was involved in data collection and made valuable contributions to the manuscript. CT, CL, JT were involved in data collection and made valuable contributions to the manuscript. PC was involved in data analysis and made valuable contributions to the manuscript. All authors read and approved the final manuscript.
